# Constitutive Law Identification and Fatigue Characterization of Rigid PUR Elastomers 80 ShA and 90 ShA

**DOI:** 10.3390/ma15196745

**Published:** 2022-09-28

**Authors:** Krzysztof Junik, Grzegorz Lesiuk, Szymon Duda, Krzysztof Jamroziak, Wojciech Błażejewski, Paweł Zielonka, Tomasz Socha, Arkadiusz Denisiewicz, Krzysztof Kula, Anna Szczurek

**Affiliations:** 1 Department of Mechanics, Materials Science and Biomedical Engineering, Faculty of Mechanical Engineering, Wroclaw University of Science and Technology, Smoluchowskiego 25, 50370 Wrocław, Poland; 2 The Faculty of Civil Engineering, Architecture and Environmental Engineering, University of Zielona Góra, ul. prof. Z. Szafrana 1, 65516 Zielona Góra, Poland

**Keywords:** hardness, polyurethane, fatigue, numerical analysis

## Abstract

This paper presents the results of a study of polyurethane rigid (PUR) elastomers in terms of the constitutive law identification, and analyses the effect of polyurethane elastomers’ hardness on fatigue properties. The research objects were PUR materials based on 4,4′-diphenylmethane diisocyanate (MDI) with the hardness of 80 ShA and 90 ShA, typically used in various industrial applications. Based on the performed experimental campaign under static and cyclic loading, the constitutive model proposed by Ogden is most appropriate. In addition, a hybrid numerical–experimental analysis (using FEM-DIC) of diabolo specimens’ behaviour is carried out in fatigue tests. Based on the performed fatigue test, it is worth noting that the energy approach describes the fatigue process synonymously compared to the displacement or strain approach. Finally, simple fatigue characteristics were analyzed and statistically validated for both PUR material configurations.

## 1. Introduction

A material called “polyurethane” is widely used with various properties and functionalities. The number of varieties of polyurethane allows this material to be adapted to the needs and desired properties, such as stiffness and flexibility. Among the wide range of purposes, the following can be distinguished: insulation (e.g., of buildings, pipelines, household appliances), depreciation (e.g., in the furniture industry), adhesives and coatings, material for mattresses, clothing, shoe soles, rollers, tires, and auto parts. Rubber and polymeric materials are also commonly used in vehicle suspension systems mainly due to their hyperelastic characteristics, which include low weight, corrosion resistance, and a high capacity for vibration damping and energy absorption. By shaping various mechanical parts and material modifications (composites, layered structures, hybrid joints), it is possible to achieve the appropriate stiffness characteristics. Structural components made of elastomers such as polyurethane or rubber (mainly) work excellently in compression and shear stress states, because they can be easily damaged during tensile stress [1]. One of the excellent examples of structural components used in suspension systems is metal–elastomer (such as the considered PUR material) bushings, which are indirectly related to the research topic undertaken in recent papers [2,3,4]. As already mentioned, they are used to connect individual elements of the suspension system. Suspension bushings are one of the smaller components of the chassis, but they cause many problems during vehicle operation due to fatigue loading [5,6,7,8,9,10]. The contribution of the material selection for the chassis system is substantial, and the knowledge about material properties is essential in fatigue lifetime prediction. It allows for properly designing the control arms and links, and the seating of all components in the vehicle’s structure. In comparison with rubber, PUR, with its properties, seems to be ideal for use in suspension systems [2]. It retains its elastic properties at temperatures as low as −40 °C. The structure of this polymer resembles a tangled line, which becomes intertwined when stretched, making it very difficult to break. It should be noted that common polyurethane configurations found in the marketplace come in two grades [2,3,4,11]: soft rubber replacement (70–80 ShA) and hard rubber replacement (90–95 ShA).

Therefore, an essential cognitive objective of this paper is the comparative analysis of the behaviour of this material in different varieties of hardness.

As the fatigue nature of elastomers, rubber-like materials [12], are different from the microscopic perspective, an excellent review of fatigue and fatigue crack growth rate analysis was performed in the review papers [8,13]. Based on this, it can be concluded that there exists in the literature three major competitive approaches in fatigue curves description, similar to metal’s description of fatigue curves: strain-based models (strain predictor [5,14]), stress-based models (stress predictor [15,16]), and energy-based models (energy predictor [17,18]). However, based on the literature review, it can be concluded that several attempts to describe the fatigue phenomenon under displacement control mode were performed in the paper [10]. Recently, [19] summarized the experimental fatigue campaign for rubber elastomers testing strategy. Excluding the ASTM D4482 standard, volumetric geometries of specimens were successfully tested in fatigue testing: diabolo specimens/hourglass shape/3D dumbbell specimens: [20,21,22,23,24,25,26,27,28]; dumbbell, dogbone specimens: [20,21,29,30,31,32,33]; cylindrical: [34,35,36,37,38]; ring: [39]; disc-shaped: [40,41]; and thin-film: [7,42].

In general, the fatigue lifetime can be expressed as:

(1) $$N_{f}=\alpha {\left(D\right)}^{\kappa }$$

where:

α and *κ* are experimentally determined constants;
*D*—specific damage parameter (predictor).

As reported in various experimental papers, the displacement-controlled experiment provides reliable fatigue data values [10,43,44]. An analysis of the literature reveals a significant lack of evaluation of the effect of PUR elastomeric material hardness on fatigue properties. Therefore, the main objective is to evaluate the fatigue properties of this material in the range of Shore 80 and 90 hardness. The selection of these levels of hardness is motivated by practical purposes. The PUR-type materials of 80 ShA hardness are an interesting alternative to rubber in automotive applications, assuming the conventional operation of automotive vehicles. On the other hand, materials of 90 ShA hardness are offered to the automotive market as stiffer and intended for racing applications. From an application perspective, the issue of the impact of hardness is becoming of interest to the automotive industry and other industries.

Considering the above, this article fills a gap in the results of fatigue studies of polyurethanes with two different hardness levels of 80 and 90 ShA.

## 2. Materials and Methods

The objects of interest were the two groups of polyurethane materials with levels of hardness of 80 ShA and 90 ShA. Duroplastic polyurethane (based on MDI for methylene diphenyl 4,4′-diisocyanate) was obtained by casting in an automated molding unit—a compound with proprieties fulfilling the designer’s requirements is formed from several mixed compounds. The resulting mixture is then cast into a preheated mold, and left with the mix in an oven to cure. The curing time depends on the degree of hardness required. After that, the component is demolded and the product is removed from the mold, before being returned to the oven for about 12 h for annealing. This stage is followed by mechanical processing of the over-molded parts, i.e.: turning and grinding until the final product is obtained. The study identified the target structure of the polyurethane chain and individual bonds by Raman spectroscopy.

To reveal the differences in the chemical composition of the two types of polyurethane samples, the Raman spectroscopy measurements were carried out using a Raman spectrometer LabRAM HR800 Horiba Jobin Yvon (Kyoto, Japan). The sample excitation was provided with a He–Ne laser source working at 632.8 nm. The measurements were performed in a 4000–10 cm^−1^ region with a spectral resolution of 2.5 cm^−1^. The received spectra were processed with the extraction of the background.

### 2.1. Static Tensile Tests and Numerical Identification of Constitutive Law

Static tensile testing is one of the primary testing methods for determining the basic mechanical properties of structural materials. During the test, the material’s response, in the form of elongation, to a given tensile load propagating at a constant rate is recorded. Specimens used for this type of test, as well as the test process itself for elastomers, are standardized and described in ASTM D412. The test was prepared following the mentioned standard.

The finite element method is applied to deliver strain–displacement curves and model parameters. Several material models were assessed, such as reduced polynomial, Arruda–Boyce, and Ogden.

The Arruda–Boyce model is expressed as:

(2) $$W=Nk_{B}t\sqrt{n}\left[\beta \lambda_{chain}-\sqrt{n}\ln\left(\frac{\sinh\beta }{\beta }\right)\right]$$

where:

*n* is the number of chain segments;
*k_B_*—Boltzmann constant;
*t*—temperature expressed in Kelvins;
*N*—the number of chains in the network of a cross-linked structure.

The Ogden [45] model is represented as:

(3) $$W=\overset{N}{\underset{i=1}{\sum }}\frac{2\mu_{i}}{\alpha_{i}^{2}}\left({\bar{\lambda }}_{1}^{\alpha_{i}}+{\bar{\lambda }}_{2}^{\alpha_{i}}+{\bar{\lambda }}_{3}^{\alpha_{i}}-3\right)$$

where *μ*_i_ and *α_i_* material constants.

For compressible materials, Bergstrom [46] added an additional component:

(4) $$W=\overset{N}{\underset{i=1}{\sum }}\frac{2\mu_{i}}{\alpha_{i}^{2}}\left({\bar{\lambda }}_{1}^{\alpha_{i}}+{\bar{\lambda }}_{2}^{\alpha_{i}}+{\bar{\lambda }}_{3}^{\alpha_{i}}-3\right)+\overset{N}{\underset{i=1}{\sum }}\frac{1}{D_{i}}{\left(J^{el}-1\right)}^{2i}$$

where:

*D_i_* denotes volumetric change parameter.

**Figure 1:**
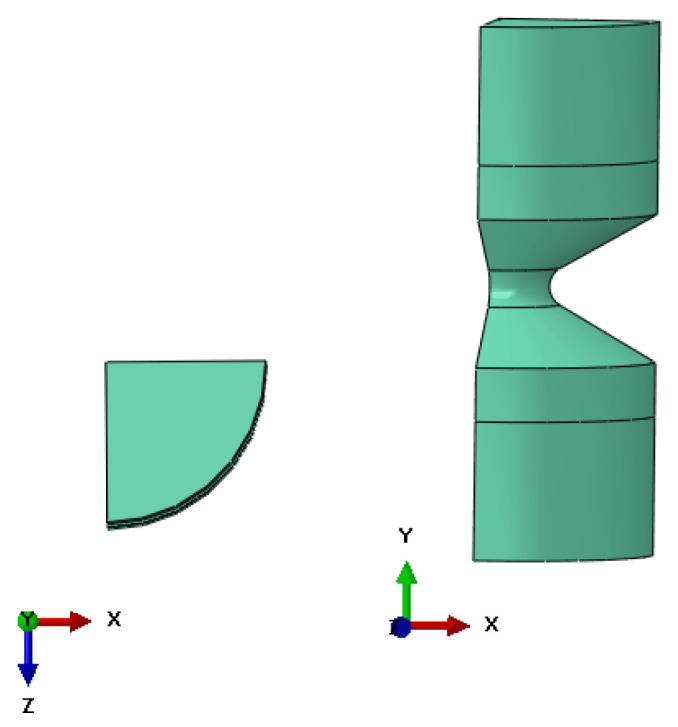
The geometry of a diabolo-shaped specimen.

The experimental data from uniaxial and planar tests were uploaded to determine model parameters. The evaluation shows that Ogden 3 parametric fits the experiment data accurately. The numerical model shows acceptable conformity with provided experiment data. A numerical model is represented by a solid geometry of a quarter of the diabolo-shaped specimen according to Figure 1. Finite element analysis was performed using Abaqus software in a static term.

**Figure 2:**
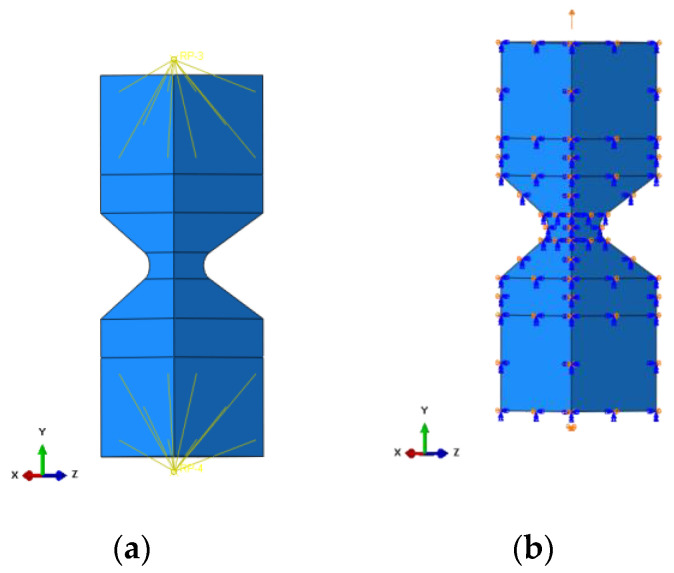
Representation of boundary conditions applied to the specimen, (**a**) coupling connection, (**b**) symmetry boundary conditions.

This modelling aims to obtain the relationship between strain and displacement for this specimen geometry. This approach allows the representation of the fatigue data in the ε–N relationship. Boundary conditions need to be applied to the geometry to provide adequate loading conditions. To keep the symmetry of the specimen, the boundaries along the x- and z-axis were provided. Transfer of the load to the sample is pursued by coupling the reference points to the inner surfaces (Figure 2a). This specific connection results in a design of the real specimen used for the fatigue test. Two reference points were used to provide fixed support (U1 = U2 = U3 = 0; translation in all three directions is pinned), and the load is supplied by a displacement boundary condition applied to the second reference point.

**Figure 3:**
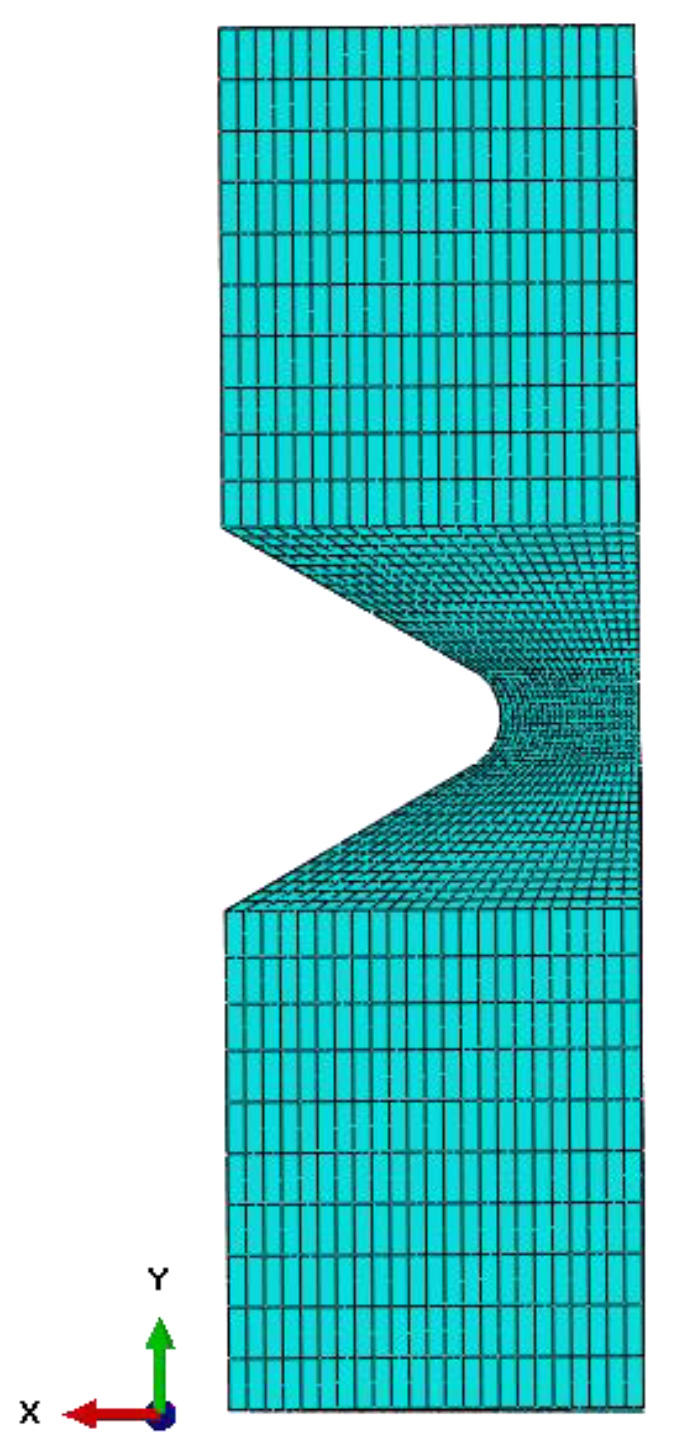
The meshed object used in FEA.

The presented geometry with the applied loading conditions meshed was into a finite continuum object. The mesh applied to the object consists of 39,402 quadratic hexahedral elements of type C3D20RH. The element size set for this model is 2 mm. However, some regions were enriched with additional nodes. Figure 3 shows the final meshed geometry; additionally, areas with the most significant number of nodes are noticeable. This enriched region allowed for obtaining more adequate results.

This finite element model was applied to investigate two material models, polyurethane 80 and 90 ShA. The simulation was run to reflect the static tensile test of this diabolo-shaped specimen and provide strain values concerning the displacement.

Additionally, the results from the FEM were assessed by applying the digital image correlation method. The Dantec Q-400 system with 2 cameras (4.8 megapixels) (Skovlunde, Denmark) was used to validate the strain pattern and value. The DIC was correlated with the FEM and the results were obtained from the Istra4D software.

### 2.2. Fatigue Tests

**Figure 4:**
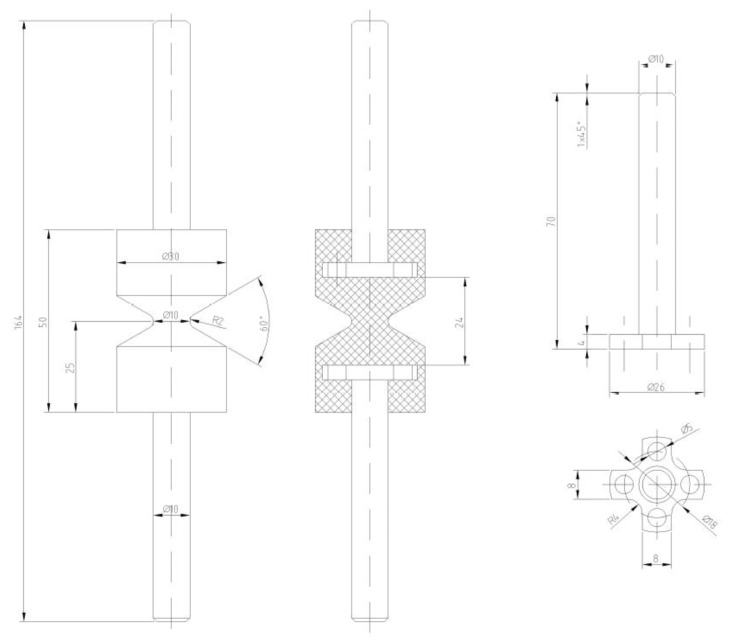
Designed diabolo specimen with metallic fixture for fatigue machine (in mm).

Fatigue tests were performed using the displacement control mode method for two displacement ratios R_d_ = 0. For the experimental campaign, a special type of specimen, diabolo, was designed, as shown in Figure 4. An essential part of the griping system was to develop a proper connection between the polyurethane specimens and metal insert.

Specimens designed in this way were manufactured by casting. Numerical analyses made it possible to determine a wide range of fatigue loads to calculate all fatigue parameters, such as stress, strain, or strain energy density, based on which fatigue diagrams were constructed afterwards.

**Figure 5:**
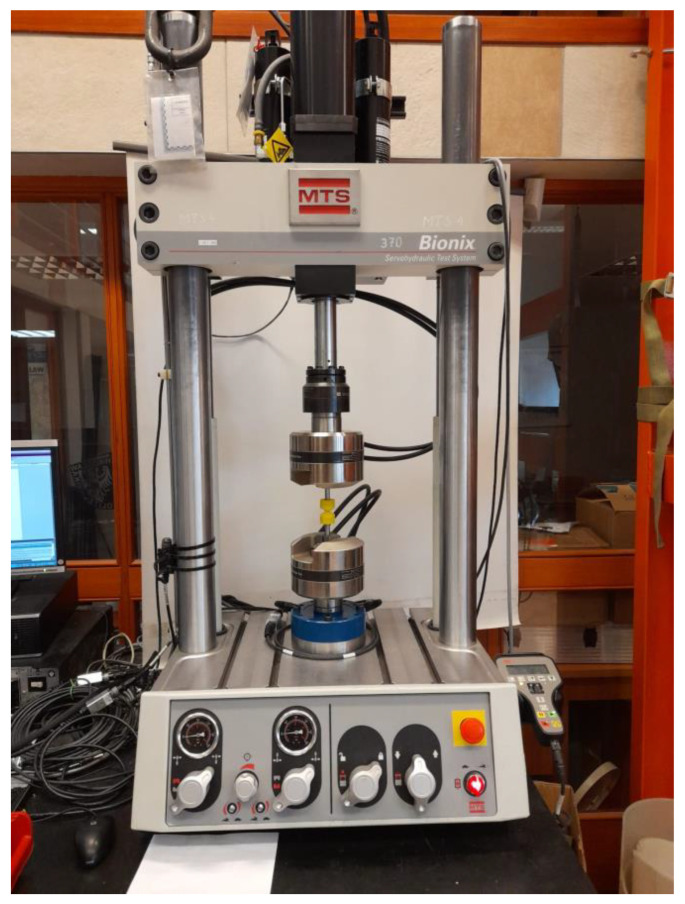
MTS858 Bionix fatigue test stand used for PUR fatigue testing.

All experiments were performed on an MTS858 Bionix (Chesterfield, MI 48051, USA) testing machine (Figure 5) with constant amplitude loads. The specimens were loaded cyclically at a frequency of 2–3 Hz. The loading frequency was chosen to avoid temperature rise during the experiment. The temperature was monitored periodically using a pyrometer. During the fatigue phase, it did not exceed a difference of 3–4 degrees Celsius. Experiments were carried out in an air-conditioned laboratory under RT conditions.

The control signal was displacement. Further, fatigue diagrams of d–N, ε–N, and W–N were constructed based on the computations, enabling a comparison of fatigue life of PUR materials with a hardness of 80 ShA and 90 ShA. A 50% decrease in specimen stiffness caused by the development of fatigue damage was used as the failure criterion.

## 3. Results and Discussion

**Figure 6:**
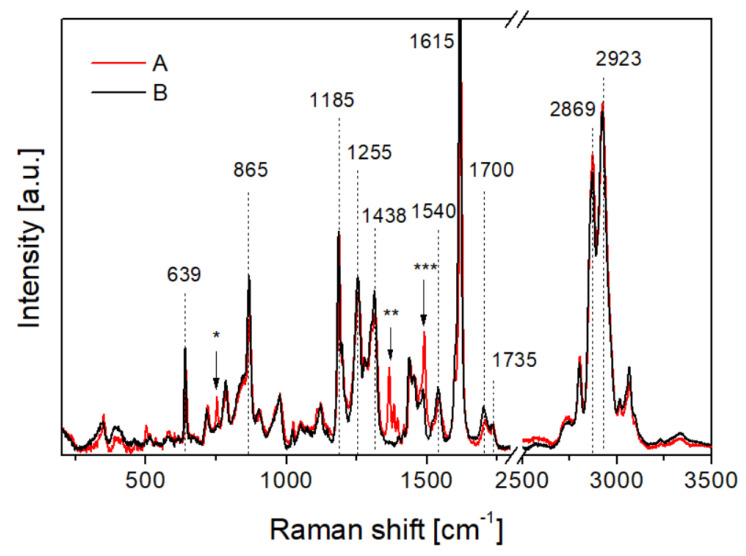
Raman spectra of polyurethanes: A—80 ShA sample, B—90 ShA sample.

Figure 6 presents Raman spectra of polyurethane samples. In spectra of both types of material, bands characteristic of polyurethane structure are observed. Bands characteristic for aromatic ring vibrations are present at 639 cm^−1^, 865 cm^−1^, and 1615 cm^−1^ [47,48,49]. Bands confirming amide groups are observed at 1255 cm^−1^ and 1540 cm^−1^, characteristic of C-N and N-H stretching of amide II [48,49,50]. Bands characteristic for N=C=O stretching and CH_2_ bending vibrations are observed at 1438 cm^−1^ [48,50]. The band observed at 1185 cm^−1^ confirms C-O-C links [51]. The band at 1700 cm^−1^ confirms the presence of hydrogen-bonded carbonyl groups (C=O), and the band at 1735 cm^−1^, free carbonyl groups [52]. In the presented spectra, the ratio of hydrogen bonded to free C=O groups is higher for the 90 ShA material. The strong bands at 2869 cm^−1^, and 2923 cm^−1^ are characteristic of CH_2_ stretching [49]. Despite the convergent chemical structure of both types of studied polyurethanes, the Raman spectra reveal chemical differences between them with bands marked with “*”, “**”, “***”. In the spectrum of the 80 ShA sample, additional bands characteristic for CH_2_ rocking at 752 cm^−1^, for CH_3_ bending as four bands in the range of 1364–1416 cm^−1^, and for CH_2_ deformation vibrations at 1490 cm^−1^ are observed. This is connected with the higher content of politetrahydrofuran, containing unbranded hydrocarbon chains used for the synthesis of 80 ShA material in comparison to the 90 ShA material. The higher amount of hydrocarbon chains constituting the soft segments [53] in the polyurethane structure in the red material, and the higher amount of hydrogen-bonded carbonyl groups constituting hard segments in the polyurethane structure in the yellow material affect the mechanical properties of polyurethanes, causing higher hardness of the yellow material.

### 3.1. Static Tensile Test Results and Constitutive Law Identification

**Figure 7:**
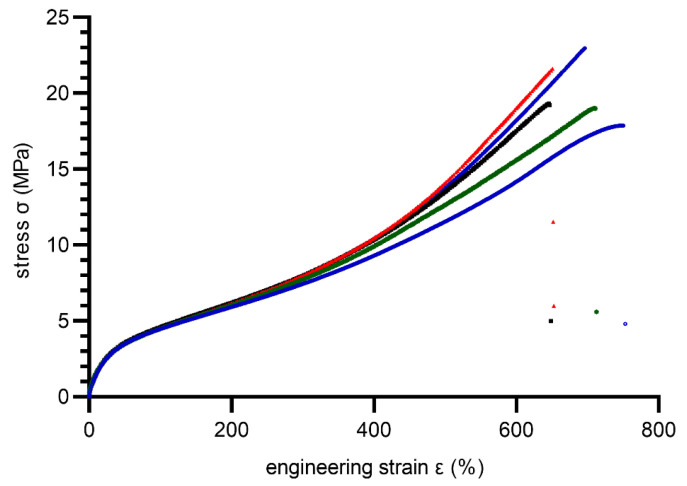
Stress–strain curves obtained during the tensile test for 80 ShA PUR material (solid lines represents different specimens, dots–measurement points after break).

**Figure 8:**
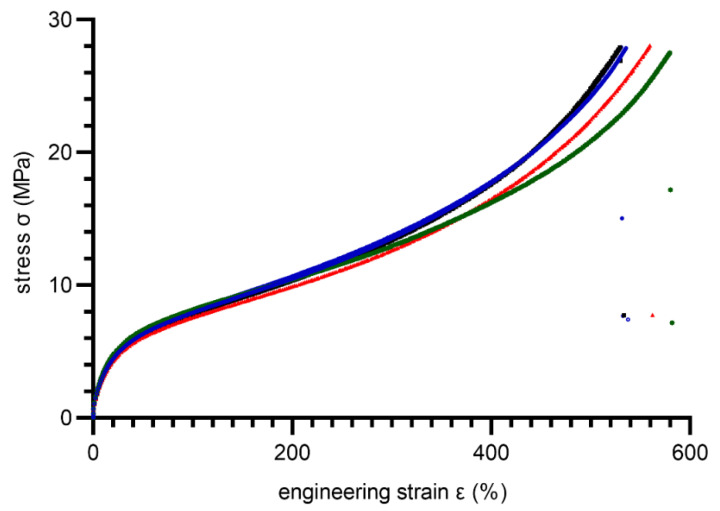
Stress–strain curves obtained during the tensile test for 90 ShA PUR material (solid lines represents different specimens, dots–measurement points after break).

In total, ten dumbbell specimens (type S1) were cut from molded plates. Before testing, all specimens were conditioned 24 h/23 ± 2 °C, 50 ± 10% RH. A tensile test was performed under displacement control mode with a rate 500 mm/min. During the test, force, displacement, and strain were measured using an extensometer for elastomeric materials. Tensile stress–strain curves are shown in Figure 7 and Figure 8 for 80 ShA and 90 ShA, respectively.

As is noticeable, the 80 ShA material exhibits larger elongation at break compared to the 90 ShA material. Summarized results of the tensile test are included in Table 1.

**Figure 9:**
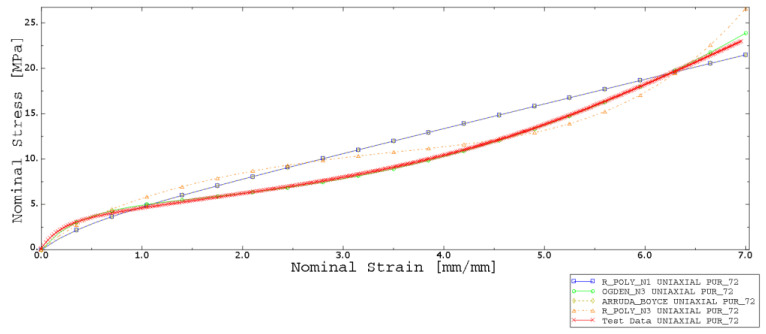
Stress–strain curves—experimental (red curve—test data) and numerical data fitting (Ogden, Arruda–Boyce, and reduced polynomial with 1 and 3 model parameters) for 80 ShA.

**Figure 10:**
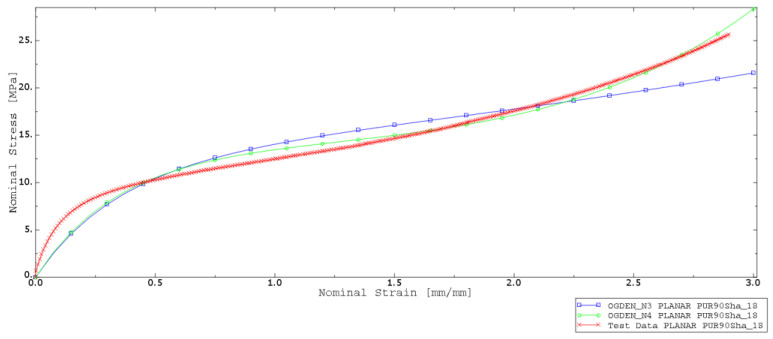
Stress–strain curves—experimental (red curve—test data) and numerical data fitting (Ogden, Arruda–Boyce, and reduced polynomial with 1 and 3 model parameters) for 90 ShA.

Abaqus CEA software allows for assessing the hyperelastic models by checking the stability of the strain in a certain range. For this purpose, two experimental data sets were provided to choose the best-fitted hyperelastic material model. The uniaxial and planar test data were counted and evaluated for choosing the more accurate model. Analysis was performed for models such as reduced polynomial, Ogden, and Arruda–Boyce, and are presented in Figure 9 and Figure 10.

Based on the performed numerical procedure, the best-fitting test was performed for the Ogden model. Obtained parameters are listed in Table 2 and Table 3.

**Figure 11:**
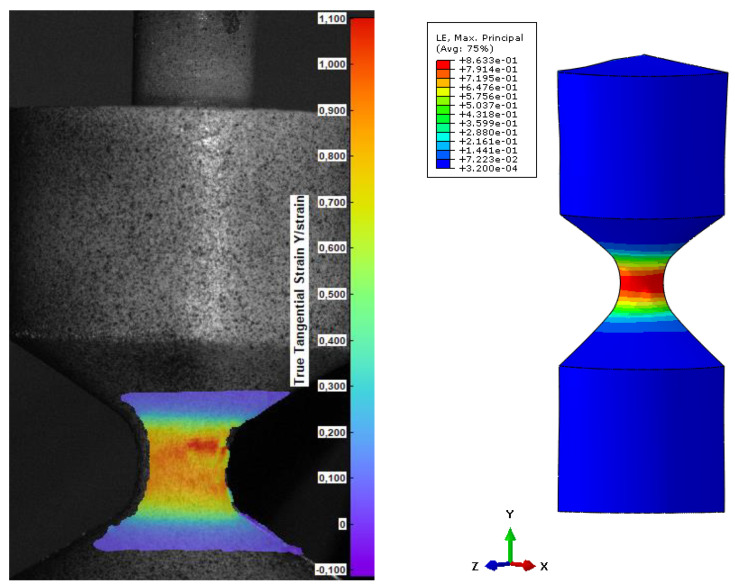
Comparison of DIC and FEM in terms of the obtained value of strain for step 60 (force DIC = 591 N, FEM force = 828 N).

**Figure 12:**
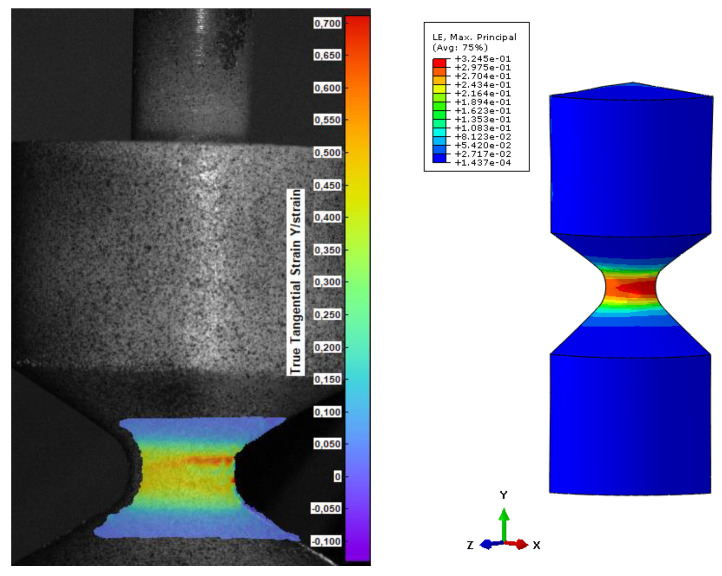
Comparison of DIC and FEM in terms of the obtained value of strain for step 30 (force DIC = 465 N, FEM force = 468 N).

**Figure 13:**
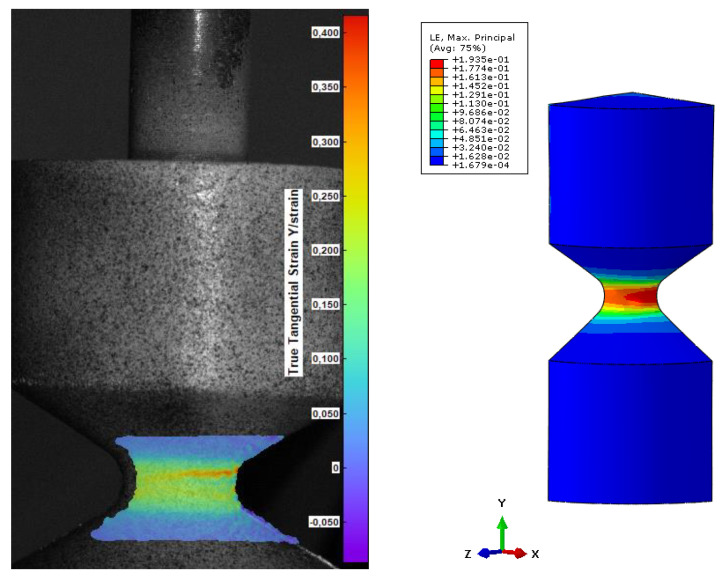
Comparison of DIC and FEM in terms of the obtained value of strain for step 15 (force DIC = 280 N, FEM force = 302 N).

To compare the results from the FEM, 3 values of strain were chosen and correlated with DIC images in terms of the reaction force acting on the geometry. The principal strain 1, which shows maximum strain for every data point mapped as a color plot on the image, was taken for steps 60, 30, and 15, and presented in Figure 11, Figure 12 and Figure 13. The comparison is based on the strain values, which is identical for DIC and FEM. Finally, the reaction force is a value that exhibits discrepancy.

According to the validation, after a certain strain level, the values (reaction force) diverge. In Figure 11, the reaction force for DIC is lower than 200 N. It is caused by incorrect adhesion along PUR and the provided pattern. Due to the high elongation of the material, the thin stochastic pattern on the surface is deboned, influencing the results of strain.

### 3.2. Fatigue Results

Fatigue experimental tests were conducted on a Bionix MTS858 machine by controlling the displacement signal. The tests were conducted on specially designed volumetric diabolo-type specimens. All results reported below are related to two hardness states, 80 ShA and 90 ShA, with a displacement ratio R = 0. The tests were conducted until the initial stiffness of the specimen drops by 50%. Typical power-law models (for displacement d, and strain ε fatigue parameters) were used in the description of the fatigue curves for better comparison with other materials available in the literature:

(5) $$d_{max}=\alpha {\left(N_{f}\right)}^{n}$$

(6) $$\varepsilon_{max}=A{\left(N_{f}\right)}^{m}$$

(7) $$W_{s}=W_{0}{\left(N_{f}\right)}^{\gamma }$$

where:

*α A*, *m*, *n*, *W*_0_, *γ*—experimentally determined constants.

**Figure 14:**
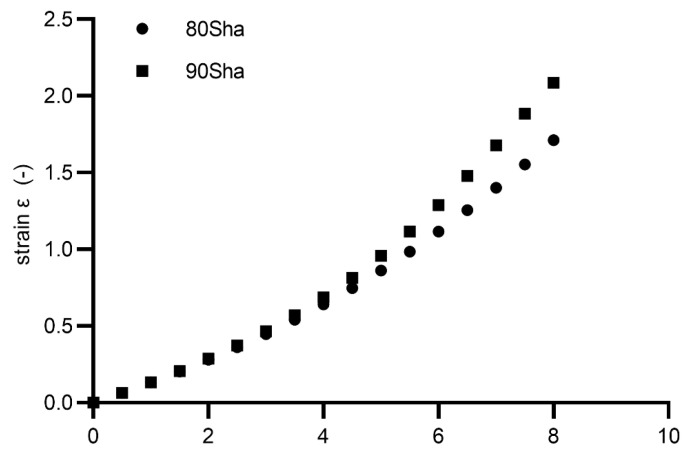
Nominal strain vs. displacement for diabolo specimens used in the experimental campaign.

Due to the complexity of the diabolo-type specimen shape, and the difficulty of directly measuring strains with the typical strain gauge, extensometer-type sensors are used. The relationship between displacement and maximum principal strain in the specimen was calibrated based on the DIC-FEM analyses performed in the previous chapters. In this way, it was possible to proceed with the construction of fatigue diagrams. Figure 14 shows the calibration curve of the strain–displacement relationship that was controlled during the experiment.

**Figure 15:**
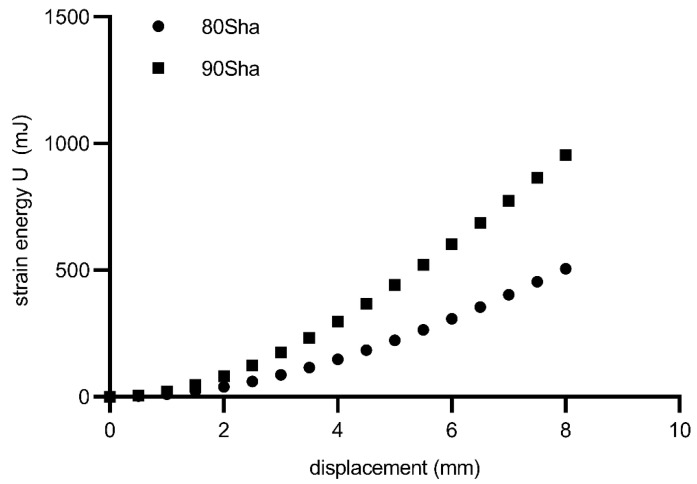
Energy vs. displacement for diabolo specimens used in the experimental campaign.

**Figure 16:**
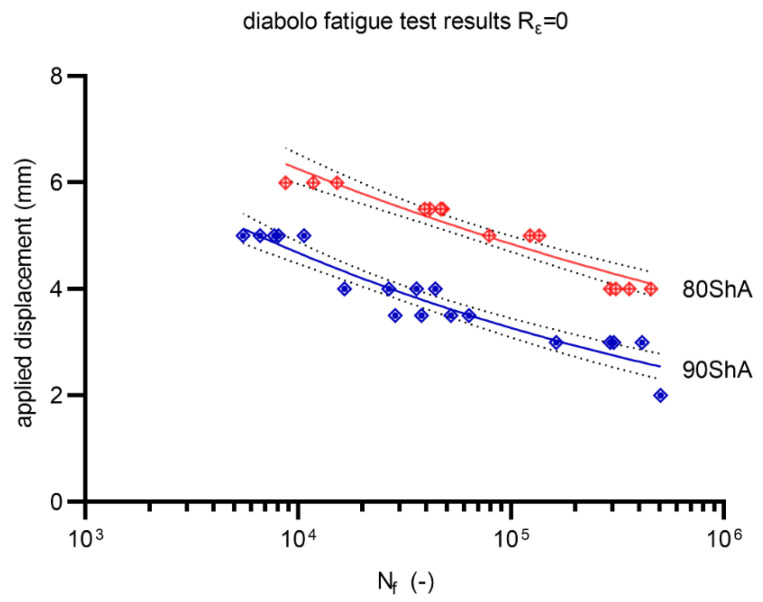
Displacement control mode fatigue data for R = 0 (80 ShA and 90 ShA).

**Figure 17:**
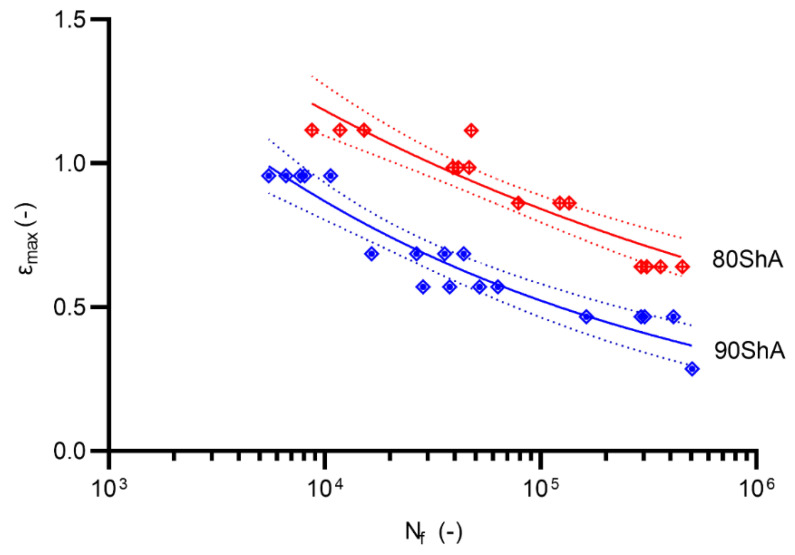
Fatigue data for R = 0 represented by *ε_max_*-N approach (80 ShA and 90 ShA).

**Figure 18:**
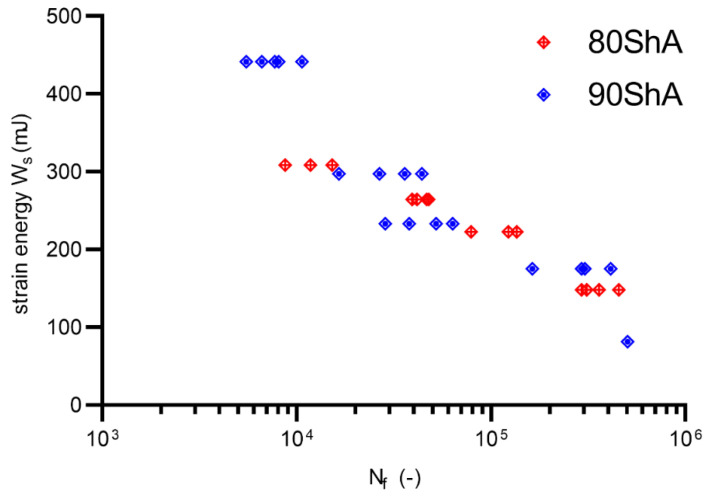
Fatigue data for R = 0 represented by *W_s_*–N energy approach (80 ShA and 90 ShA).

Similarly, a calibration between the energy parameter U was performed based on numerical calculations. The calibration results are shown in Figure 15. Fatigue curves for the 80 ShA and 90 ShA materials for the initial control signal, displacement, are shown in Figure 16. The 95% confidence intervals are also marked on this diagram. The results show that the differences in fatigue life levels are statistically significant. A similar phenomenon is observed when the quantity describing the fatigue process is strain, shown in Figure 17. On the other hand, the question arises whether, in describing elastomeric materials, assuming such quantities as strain or stress, by directly reproducing the description of the fatigue phenomenon observed in metals, is appropriate. In this case, these characteristics are significantly different in the tensile test. Therefore, a universal quantity such as strain energy was used—Figure 18. Energy as a criterion quantity dimensionally combines force and displacement, which, in the engineering sense, allows us to consider both together in describing the phenomenon.

Statistical fitting and analyses were performed in Graphpad PRISM environment. All results are collected in Table 4.

The energy description also results in a better fit of the curves, as evidenced by the greater or equal R^2^ values obtained for the underlying energy model compared to the displacement or strain models. Energy as a criterion quantity unambiguously describes the fatigue process, and further observations in the field of PUR materials modeling should be performed from the energy perspective.

## 4. Conclusions

All the research and analysis conducted on PUR materials in the hardness range from 80 ShA and 90 ShA allows us to draw the following conclusions:

- Ogden’s model provides the best description of the behavior of polyurethane material based on MDI for methylene diphenyl 4,4′-diisocyanate for both hardness types in the range of 80 ShA and 90 ShA;
- The higher content of politetrahydrofuran containing unbranded hydrocarbon chains is characteristic for 80 ShA material compared to the 90 ShA material—this allows for the mechanical properties of both materials to change in the static range in the sense of stress–strain curves in uniaxial tensile testing. The 90 ShA material is characterized by higher stiffness and stress levels characteristic of 100%, 200%, and 300% strain levels;
- Regarding strain approach, the fatigue process of PUR elastomers for 80 ShA and 90 ShA hardness show statistically significant differences. Under displacement control mode, the 80 ShA material exhibits an apparently higher fatigue strength;
- The fatigue process analysis from the strain energy point of view makes it possible to describe it unambiguously. In the case analyzed, there are no significant differences in the *W–N* fatigue curves for the 80 ShA and 90 ShA hardness levels;
- The energy approach is also characterized by a better statistical fitting of the measurement data to the energy model. It enables the accurate prediction of the fatigue life of components, regardless of the ranges and types of loads during fatigue tests based on the force or displacement control mode of fatigue experiments.

## Figures and Tables

**Table 1:** Tensile test results analysis for 80 ShA and 90 ShA material configuration.

Specimen ID	UTS—Ultimate Tensile Strength in MPa	A—Elongation at Break In %
PU80_#1	17.9	749.2
PU80_#2	19.4	646.4
PU80_#3	21.6	651.0
PU80_#4	19.0	710.4
PU80_#5	23.7	711.0
PU80 (median ± std.dev)	19.4 ± 2.3	710.4 ± 43.9
PU90_#1	28.0	530.9
PU90_#2	27.9	529.5
PU90_#3	28.1	559.6
PU90_#4	27.5	579.5
PU90_#5	27.9	535.3
PU90 (median ± std.dev)	27.9 ± 0.2	535.3 ± 21.9

**Table 2:** Ogden model parameters for 80 ShA.

N	$\mu_{i}$	$\alpha_{i}$
1	4.34400372	−0.380731347
2	0.210081339	3.47276528
3	4.921467147 × 10^−3^	6.93803394

**Table 3:** Ogden model parameters for 90 ShA.

N	$\mu_{i}$	$\alpha_{i}$
1	−707.693690	1.13390568
2	316.165559	1.34789067
3	401.397194	0.904727974
4	8.765965905 × 10^−3^	−7.00870716

**Table 4:** Fatigue models data fitting for 80 ShA and 90 ShA.

	*α* (mm)	*n* (−)	R^2^	*A* (−)	M (−)	R^2^	*W*_0_ (mJ)	*γ* (−)	R^2^
80 ShA	17.32	−0.1106	0.91	4.627	−0.148	0.87	1870	−0.189	0.91
90 ShA	19.62	−0.1556	0.90	6.592	−0.2201	0.91	5362	−0.283	0.92
